# Activated Porous Carbon Derived from Tea and Plane Tree Leaves Biomass for the Removal of Pharmaceutical Compounds from Wastewaters

**DOI:** 10.3390/antibiotics10010065

**Published:** 2021-01-11

**Authors:** Efstathios V. Liakos, Kyriazis Rekos, Dimitrios A. Giannakoudakis, Athanasios C. Mitropoulos, Jie Fu, George Z. Kyzas

**Affiliations:** 1 Department of Chemistry, International Hellenic University, 65404 Kavala, Greece; stathilas@gmail.com (E.V.L.); amitrop@chem.ihu.gr (A.C.M.); 2 Department of Chemistry, Aristotle University of Thessaloniki, 54124 Thessaloniki, Greece; rkyriazis@chem.auth.gr; 3 Institute of Physical Chemistry, Polish Academy of Sciences, Kasprzaka 44/52, 01-224 Warsaw, Poland; dgiannakoudakis@ichf.edu.pl; 4 School of Environmental Science and Engineering, Huazhong University of Science and Technology, Wuhan 430074, China

**Keywords:** activated carbons, adsorption, pharmaceuticals, agricultural wastes

## Abstract

The aim of the present study is the synthesis of activated carbon (AC) from different agricultural wastes such as tea and plane tree leaves in order to use them for the removal of pramipexole dihydrochloride (PRM) from aqueous solutions. Two different carbonization and synthetic activation protocols were followed, with the herein-proposed ultrasound-assisted two-step protocol leading to better-performing carbon, especially for the tea-leaf-derived material (TEA(char)-AC). Physicochemical characterizations were performed by Fourier-transform infrared spectroscopy (FTIR), N_2_ physisorption, and scanning electron microscopy (SEM). TEA(char)-AC presented the highest surface area (1151 m^2^/g) and volume of micro and small mesopores. Maximum capacity was found at 112 mg/g for TEA(char)-AC at an optimum pH equal to 3, with the Langmuir isotherm model presenting a better fitting. The removal efficiency of TEA(char)-AC is higher than other biomass-derived carbons and closer to benchmark commercial carbons.

## 1. Introduction

Water is one of the most precious resources on Earth since it is the foundation of every kind of life. Additionally, aquatic resources are essential for societies and economies, from utilization for agricultural and manufacturing needs to power generation [1]. Pollution of available aquatic resources is assumed to be one of the greatest threats arising from continuous modernization and technological evolution that consequently jeopardizes water availability in the future. Various organic compounds are regarded nowadays as emerging pollutants since they are used in a plethora of every-day life activities and can be discharged to aquatic environments in various ways, like improper and uncontrolled disposal from industrial, agricultural, and municipal occupations [2]. Organic compounds that are already well known to pose an environmental threat are detergents, personal care products, fragrances, flavorants, dyes, and many more [3,4,5,6,7]. Another category of organic pollutants that are important to modern societies is pharmaceuticals; they are disposed of in an uncontrolled manner, reaching groundwater and soil, and they remain persistent [8]. Since chronic exposure to pharmaceuticals may produce unknown toxicological effects, they are regarded as dangerous environmental contaminants [9,10,11]. It is a fact that the presence of pharmaceuticals in wastewater effluents and surface waters is a potential threat to human health, ecosystems [12], and the quality of drinking water [13]. These pharmaceutical compounds have been hugely overused due to rapid urbanization, overpopulation, and climate change and are consumed by industry and humans [14]. As a result, these pharmaceutical compounds harm the environment because, in many cases, they are directly discharged into water reservoirs or resources [15,16]. It is a major priority to detect and face the problem of emerging pollutants from wastewater treatment plants (WWTPs). There are numerous studies that have presented this type of data, and, indeed, the occurrence of pharmaceuticals as emerging pollutants is a big problem. Some examples are paraxanthine, caffeine, acetaminophen, atenolol, metoprolol, propranolol, erythromycin, sulfamethoxazole, trimethoprim, diclofenac, indomethacin, ketoprofen, mefenamic acid, and carbamazepine [17,18].

For the removal of pharmaceuticals from aquatic systems, groundwaters, industrial and hospital wastewaters as well as drinking-water treatment plants, a plethora of technologies and methods have been explored, including ion exchange, coprecipitation, filtration, reverse osmosis, Fenton oxidation, biodegradation, ozonation, and electrochemical reduction. The main disadvantages of these technologies are the economic aspects and complexities of real-life application and, in some cases, the necessity of use of other, often hazardous, chemical reagents, leading to nonecological friendly processes. On the contrary, adsorption-based remediation methods are more compatible with the principles of green chemistry and sustainable development. Additionally, adsorption processes are assumed as effective, versatile, and economically feasible.

The most crucial aspect of effective adsorptive remediation applications is the utilized adsorbent [19,20,21,22,23]. Activated carbons (ACs) are considered among the best candidates. The structure of activated carbon can be separated into micropores, mesopores, and macropores, with pore widths less than 2 nm, between 2 and 50 nm, and greater than 50 nm, respectively [24]. The skeleton of nanoporous carbon consists mainly of carbon atoms (major element) that are overlapped with hydrogen and oxygen groups, the percentage of which is dependent on the carbonaceous precursor. Additionally, according to the synthesis route, postsynthesis functionalization may also contain phosphorus-, nitrogen-, or sulfur-containing groups. In addition, it must be noted that these heteroatoms are mainly located on the edges of the basal planes, a phenomenon that is attributed to the presence of highly reactive unsaturated carbon atoms. However, the oxygen surface groups present have, by far, the highest percentage. Additionally, the activated carbon materials may also contain inorganic compounds due to the ash content (sometimes 20% wt.), which is mainly generated from the nature of the selected carbonaceous precursor [24]. Activated carbons present good properties due to their relatively low-cost and availability, their promising textural features, and tuneable surface chemistry heterogeneity [25,26,27,28,29,30,31,32]. However, a disadvantage exists and is linked with the raw precursor from which ACs are synthesized, coconut shell, coal, and wood being the predominant feedstocks [33]. Consequently, in many places, these precursors must be imported, and, in addition to the fact that their price is quite high, their transportation also has a negative impact on the carbon dioxide footprint. Hence, this major aspect should be considered when designing novel ACs—to use locally available abundant resources. The use of biomass discarded during the production of valuable goods, especially agricultural ones, can have a beneficial role in cycle economy sustainability. Tea is a popular beverage on a worldwide scale. In 2015, the production of tea in China was estimated to be 2.27 million tons, representing 40% of worldwide output. The gradual increase in the global consumption of tea, in addition to the high demand for tea-derived products, has generated a large quantity of tea wastes [34]. For the synthesis of activated carbons (ACs), two processes are involved for carbonization and activation—the chemical and the physical. Usually, physical activation includes the carbonization (400–600 °C) [35] of a carbonaceous precursor under an inert atmosphere (N_2_) and activation (800–1000 °C) under oxidizing gases (CO_2_, steam, and mixtures of them) [36], while chemical reagents are used in the case of chemical activation.

In this study, the goal is to optimize the synthesis of the tea-waste-derived activated carbon and, especially, tea and plane tree leaves and to explore the potential of utilizing them as efficient adsorbents in aqueous environments against a pharmaceutical compound, pramipexole dihydrochloride. Regarding AC synthesis, the first synthetic protocol involves two thermal treatment steps under an N_2_ inert atmosphere, with the ultrasound-assisted impregnation of NaOH as the activation occurring between the two thermal treatment steps. The second synthesis is a classic one-step carbonization/activation using the same activation agent under the same atmosphere. For the sake of comparison, tea and plane tree leaves were used as the precursor biomass.

## 2. Results and Discussion

### 2.1. Characterizations

#### 2.1.1. N_2_ Physisorption Tests

**Figure 1:**
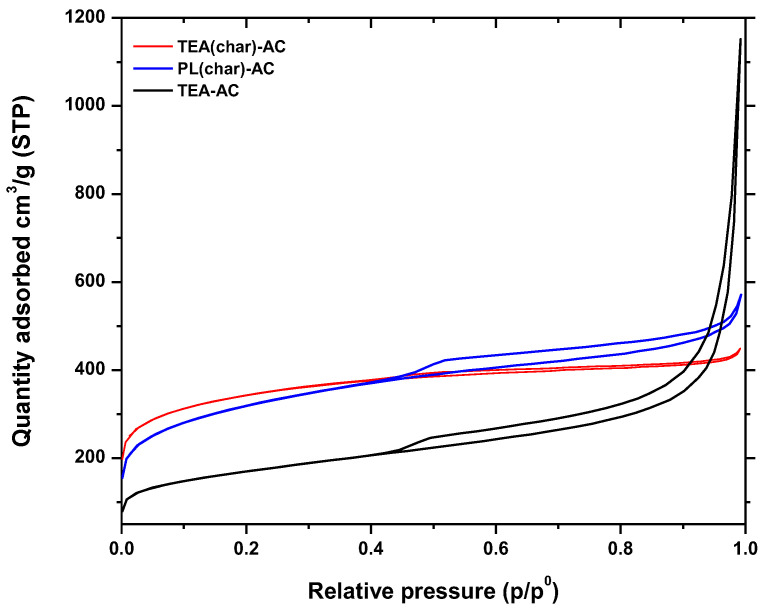
N_2_ physisorption isotherms of the synthesized activated carbon (AC) samples.

The nitrogen adsorption/desorption isotherms for the prepared ACs are presented in Figure 1. According to IUPAC classification, the adsorption isotherms of TEA(char)-AC, PL(char)-AC, and TEA-AC are revealed as a combination of Type I (for low values of relative pressure p/p^0^) and Type IV (for higher relative pressure), indicating the combined microporous and mesoporous nature [37]. These results were also confirmed due to the presence of H_3_ and H_4_ desorption hysteresis loops that are characteristic of microporous and mesoporous carbons [38,39].

In particular, in the case of microporous materials, the BET method can be applied to many Type II and Type IV isotherms, but extreme caution is needed in the presence of micropores (i.e., with Type I isotherms and combinations of Types I and II or Types I and IV isotherms). It may be impossible to separate the processes of monolayer and multilayer adsorption and micropore filling. With microporous adsorbents, the linear range of the BET plot may be very difficult to locate [38,39]. There is a useful procedure [40] that allows us to overcome this difficulty and avoid any subjectivity in evaluating the BET monolayer capacity. This procedure is based on the following main criteria: (a) quantity C should be positive (i.e., a negative intercept on the ordinate of the BET plot is the first indication that one is outside the appropriate range); (b) application of the BET equation should be restricted to the range where the term n(1 − p/p^0^) continuously increases with p/p^0^; (c) the p/p^0^ value corresponding to nm should be within the selected BET range. It must be re-emphasized that this procedure should not be expected to confirm the validity of the BET monolayer capacity. Thus, the BET area derived from a Type I isotherm must not be treated as a realistic probe-accessible surface area. It represents an apparent surface area, which may be regarded as a useful adsorbent “fingerprint”.

The textural features collected in Table 1 clearly show that TEA(char)-AC has the highest specific surface area (1151 m^2^/g), while PL(char)-AC and TEA-AC were approximately 4% and 48% lower. TEA-AC presented the highest total pore volume (V_Tot_) of 1.782 cm^3^/g and mesopore volume of 1.703 cm^3^/g. PL(char)-AC revealed approximately 27.4% higher V_Tot_ comparing to TEA(char)-AC and the highest external surface area (597 m^2^/g).

**Figure 2:**
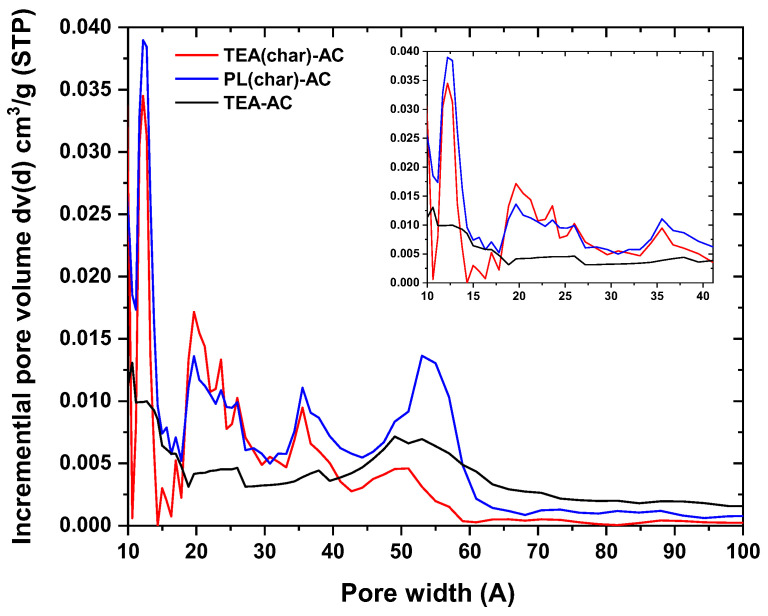
Pore size distributions of the synthesized AC samples.

An interesting outcome can be derived from the comparison of the micropores-to-mesopores ratio, with TEA(char)-AC showing the highest ratio by far. An important aspect of microporous adsorbent characterization is the determination of their pore size distribution (PSD). The most widely used methods are the density functional theory (DFT) and the Horvath–Kawazoe equation [41]. Here, the DFT method was developed for activated carbons with simple slit geometries and the so-called nonlocal-density functional theory (NLDFT) [42]. In the present study, a pore size distribution analysis (Figure 2) revealed that the two-step synthesis led to a higher volume of pores within the range of micropores to small mesopores. These pores are well known to play a key role in the removal efficiency of activated carbon for organic molecule adsorption (e.g., dye molecules, pharmaceutical compounds) [43,44,45]. All the materials were shown to have pores from 4 to 6 nm, which is crucial for the diffusion and adsorption of the pollutants onto active adsorption centers or sites [46,47,48].

#### 2.1.2. SEM Images

**Figure 3:**
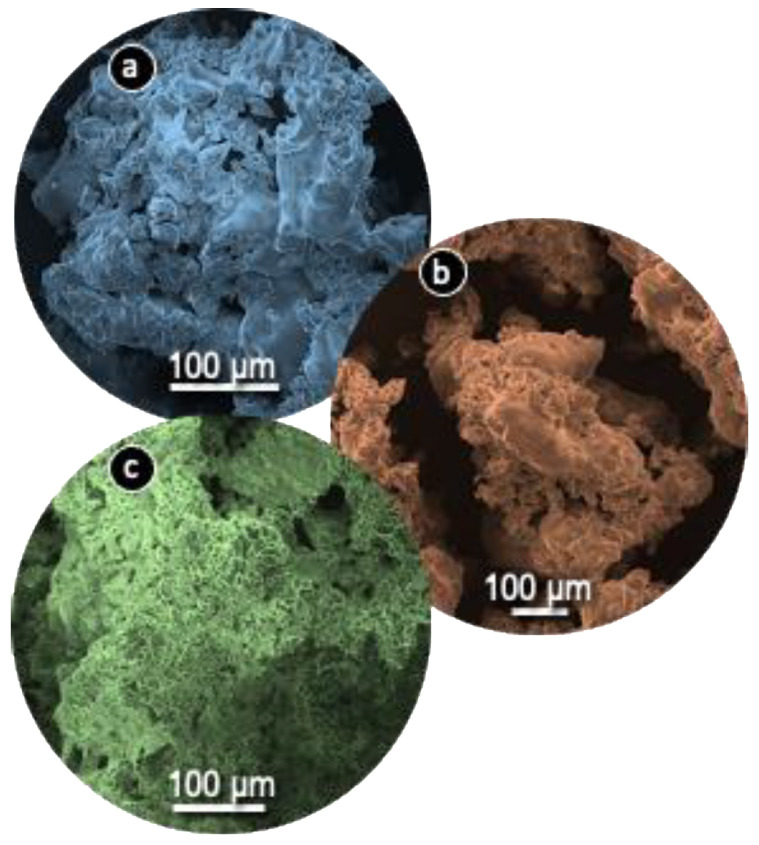
SEM images of tea-leaf-derived material (TEA(char)-AC) (**a**), plane-tree-leaf-derived material (PL(char)-AC) (**b**), and TEA-AC (**c**).

The SEM images (Figure 3) showed a rough morphology for all AC samples. This fact is attributed to the intercalation of sodium salts in the structure of the carbonaceous source [49].

During pyrolysis, volatiles are generated from the impregnated carbonaceous surface, which results in a porous network. Specifically, the cavities are produced from the space that was previously occupied by sodium salts due to the impregnation process, which thereafter, were removed due to the process of pyrolysis. As can be observed from the SEM images, TEA(char)-AC and PL(char)-AC have similar structures, with a plethora of big size voids, in contrast with TEA-AC, which has a more condensed structure due to the differently followed synthesis route. The less-dense nature can have a beneficial impact on faster adsorption.

### 2.2. Adsorption Evaluation

#### 2.2.1. Effect of pH–FTIR Explanations

**Figure 4:**
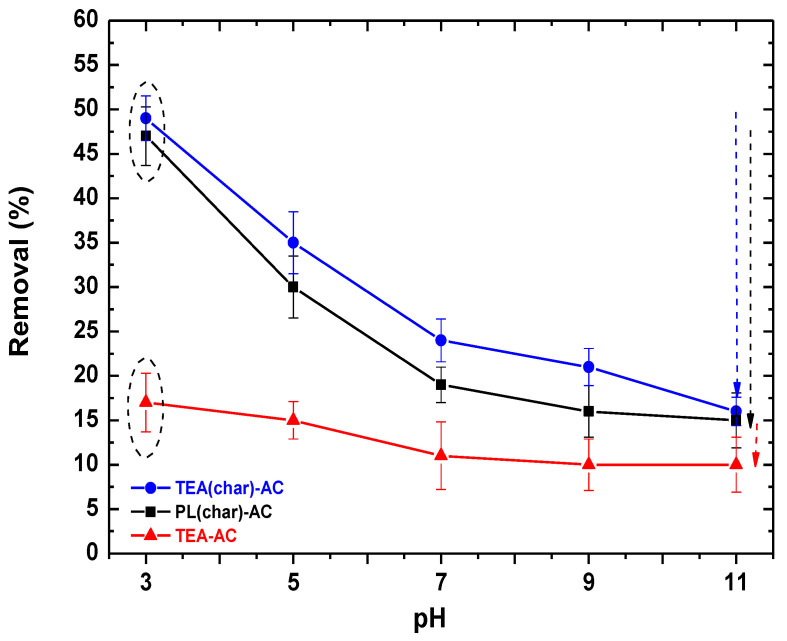
pH effect for the removal of PRM from the liquid phase.

Figure 4 depicts the removal efficiencies of the synthesized ACs under various initial pHs, ranging from 3 to 11. For all samples, the optimum value of pH was found to be 3, in good correlation with other reports in the literature [43], reaching 49%, 47%, and 17% RPM removal for TEA(char)-AC, PL(char)-AC, and TEA-AC, respectively. The removal of PRM decreases with the increment of the initial pH for all types of AC samples (TEA(char)-AC: from 36% (pH 5) to 24.5 (pH 7), and finally 15.5 (pH 11); PL(char)-AC: from 31 (pH 5) to 20 (pH 7), and finally 15 (pH 11); TEA-AC: from 16% (pH 5) to 12% (pH 7), and finally 10% (pH 11). The optimum value of pH was found to be 3 for all cases, a result that is similar to another study.

**Figure 5:**
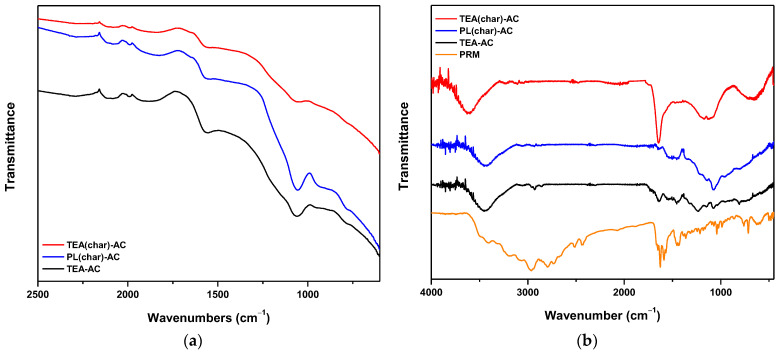
FTIR spectra of all studied samples (**a**) before adsorption and (**b**) after pramipexole dihydrochloride (PRM) adsorption.

To explain adsorption interactions, it is mandatory to analyze FTIR spectroscopy before and after PRM adsorption. Figure 5a depicts the FTIR spectra for all synthesized AC samples before adsorption. TEA(char)-AC, PL(char)-AC, and TEA-AC samples showed a very weak band at 2851, 2854, and 2870 cm^−1^, respectively, which are attributed to the C–H stretching vibrations due to aliphatic moieties. The bands at 1630, 1632, and 1659 cm^−1^ are attributed to the carbonyl group (C=O) axial deformation in the case of TEA(char)-AC, PL(char)-AC, and TEA-AC samples, respectively. Additionally, very weak bands are recorded at 1480, 1487, and 1500 cm^−1^ for TEA(char)-AC, PL(char)-AC, and TEA-AC samples, respectively, due to the stretching vibration of C=C bonds [50]. The bands at 1059, 1060, and 1075 cm^−1^ are recorded (for TEA(char)-AC, PL(char)-AC, and TEA-AC samples, respectively) due to the C–O stretching vibrations bonds of esters, phenols, alcohols, or ethers [50,51]. In the case of PL(char)-AC and TEA-AC, the very weak bands at 580 and 584 cm^−1^ are attributed to O–H out-of-plane bending vibrations, respectively [50].

**Figure 6:**
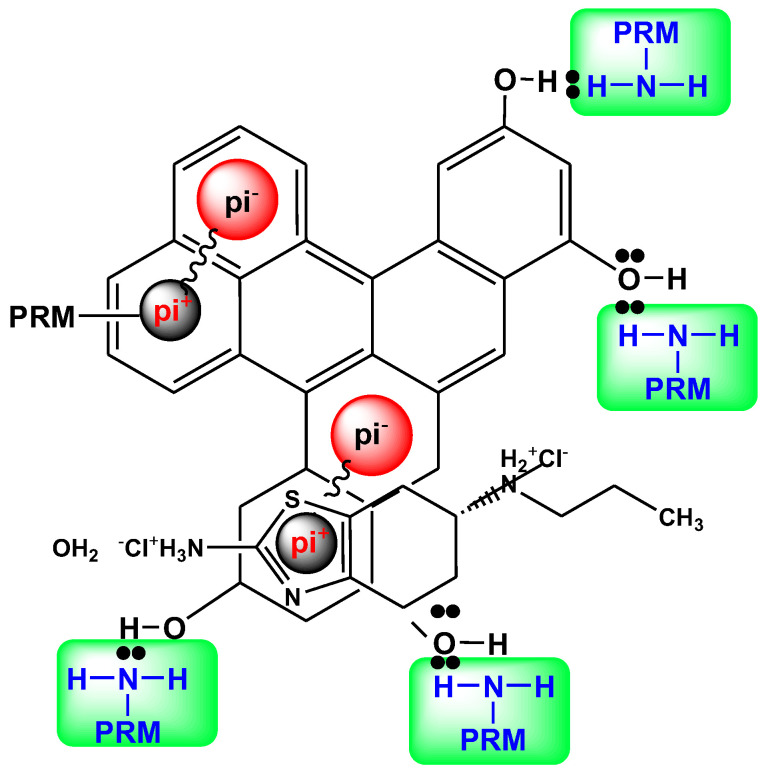
Possible interactions between PRM molecules and activated carbons (ACs).

Figure 5b shows the FTIR spectra after the adsorption of PRM. The band at 1632 cm^−1^ for TEA(char)-AC was shifted to 1685 cm^−1^, the one at 1630 cm^−1^ for PL(char)-AC to 1650 cm^−1^, and at 1659 cm^−1^ for TEA-AC to 1670 cm^−1^, indicating that the C=O functional groups were also interacting with PRM molecules. Moreover, in the case of PL(char)-AC and TEA-AC, the bands at 1480 and 1500 cm^−1^ were shifted to 1502 and 1490 cm^−1^, respectively, indicating that C=C also interacted with PRM molecules. Then, the band at 1059 cm^−1^ was shifted to 1070 cm^−1^, 1075 cm^−1^ shifted to 1050 cm^−1^, and 1060 cm^−1^ shifted to 1070 cm^−1^ (TEA(char)-AC, PL(char)-AC, and TEA-AC, respectively), indicating that the functional groups of C–O were interacting with PRM molecules, and, in the case of TEA-AC, a new band appeared at 1250 cm^−1^, which may be attributed to the C–O stretching of aromatic esters. In addition, the very weak band at 582 cm^−1^ shifted to 645 cm^−1^, 584 cm^−1^ shifted to 765 cm^−1^, and 580 cm^−1^ shifted to 763 cm^−1^ for TEA(char)-AC, PL(char)-AC, and TEA-AC, respectively, indicating that the O–H functional groups are responsible for PRM adsorption. Another worthwhile finding is that the bands at 1615 cm^−1^, presented in all ACs before adsorption, had shifted to 1585 cm^−1^, which can be majorly attributed to the dispersive interactions between the pi-electrons of the benzene rings of the PRM molecule and the electron-rich region in the aromatic ring of the activated carbon samples [43]. The main interactions are collectively illustrated in Figure 6.

Based on the above FTIR results, it can be concluded that strong pi–pi electron coupling and/or stacking (mainly dispersion forces) between PRM molecules and aromatic rings of the carbon may be the adsorption mechanism. The benzene rings of the drugs and their aromatic heterocyclic rings are expected to interact with the polarized aromatic rings on carbons via the mechanism of pi–pi electron coupling [43,52]. Additionally, the Lewis acid-base interaction, where amino groups of PRM molecules are the Lewis bases and the O-containing groups of carbons serve as Lewis acids, may be another important mechanism of adsorption of PRM. The presence of lone pairs of electrons on nitrogen atoms produces dipolar moments for PRM. Negative charges are close to these nitrogen atoms, and the presence of polar oxygen groups on the carbon surface, with lone pair of electrons on their oxygen atoms, may be the reason for surface-specific interactions between the oxygen surface groups of carbon samples and PRM molecules [43]. The latter was in accordance with another study [43], in which the adsorbent material was activated carbon produced from potato peels, and the pollutants adsorbed were pharmaceutical compounds (pramipexole and dorzolamide).

#### 2.2.2. Equilibrium Isotherms and Kinetics

**Figure 7:**
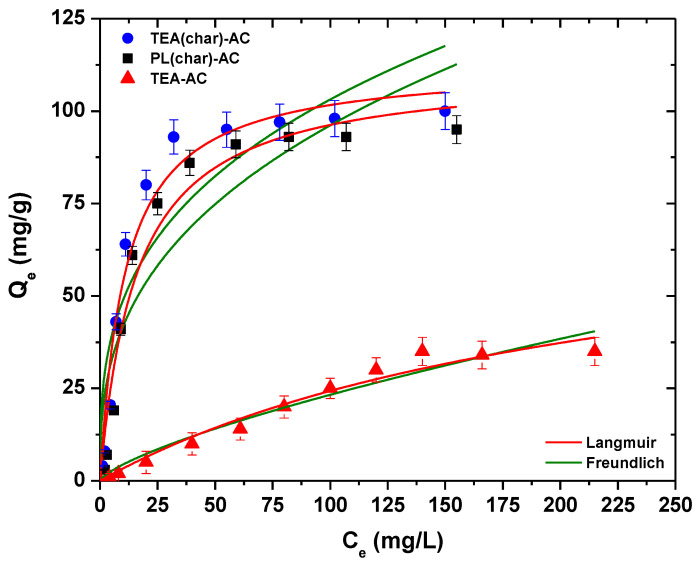
Isotherms of the synthesized AC samples at 25 °C.

Figure 7 shows the isotherms that were obtained after PRM adsorption from aqueous solutions. Table 2 collects the parameters after the fitting of experimental data to the Langmuir and Freundlich models.

The correlation coefficients (R^2^) revealed a better fitting of the Langmuir model for TEA(char)-AC and PL(char)-AC, while the Freundlich model fits better in the case of TEA-AC. Maximum capacity was found to be 112 mg/g for TEA(char)-AC, a value of about 11% higher compared to PL(char)-AC. TEA-AC showed a smaller (by around 3.3-fold) removal efficiency. In order to determine which physicochemical feature of the materials plays a key role, the capacities expressed per the specific surface area and per the total pore volume were calculated. The mg adsorbed per surface area values were found to be approximately 0.097, 0.092, and 0.057 mg/m^2^ for TEA(char)-AC, PL(char)-AC, and TEA-AC, respectively, while the mg adsorbed per total pore volume were 161, 114, and 19 mg/cm^3^. These values suggest that the surface adsorption efficiency is higher for TEA(char)-AC and lowest for TEA-AC, revealing the importance of the micropore structure associated with small mesopores that was obtained when a two-step protocol involving carbonization, followed by impregnation and activation, was followed.

**Figure 8:**
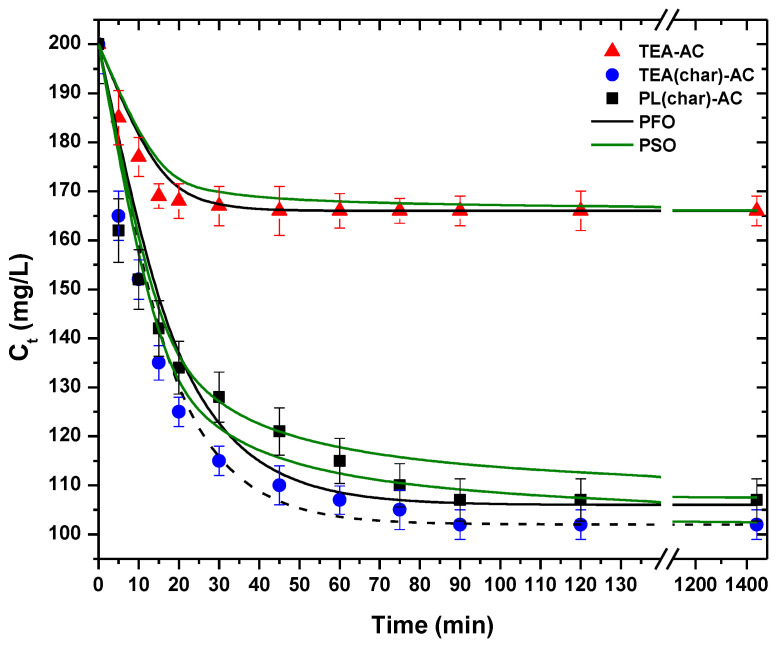
Kinetic curves for PRM adsorption onto the synthesized AC samples.

Another crucial factor for the process of adsorption is the effect of contact time. The kinetic trend is presented in Figure 8. As can be observed, all the prepared materials presented similar kinetic behavior.

Sharp PRM removal is observed at the first stage of the adsorption (5–15 min), after which a more gradual decrease of PRM concentration in the solution is observed (20–50 min) before reaching a plateau (equilibrium). The kinetic data were fitted to the PFO and PSO equations, but as can be observed from the calculated parameters (Table 3), the PFO had better fitting (R^2^_PFO_ = 0.963–0.994, R^2^_PSO_ = 0.946–0.980).

### 2.3. Comparison with Other Materials

Table 4 summarizes the highest PRM adsorption capacities (Q_max_) reported in the literature, even though a direct comparison is a complex aspect since various parameters, experimental and cost-oriented, should be considered. However, we can conclude that the biomass-derived carbons obtained by the 2-step synthesis showed capacity values close to BAX commercial carbon, which is considered a benchmark. The most important observation is that the herein-synthesized ACs showed higher removal efficiencies than other biomass-derived carbons. Chitosan showed higher capacity compared to all carbons, a fact that reveals that surface chemistry heterogeneity is more crucial than textural features. Surface functionalization of chitosan led to a significant remediation improvement. Hence, further functionalization of the herein-studied ACs can be worthwhile exploring in the future.

## 3. Materials and Methods

### 3.1. Materials

The raw biomass used for the synthesis of ACs consisted of leaves from either tea or the plane tree. The tea species used was *Camellia sinensis* (known as *Camellia sinensis* L.). Tea leaves contain caffeine, polyphenols (such as flavonoids), and epigallocatechin gallate. It is quite evident that the generation of such massive quantities of waste from the tea industry alone will undoubtedly result in a host of environmental problems associated with their improper disposal [54]. On the other hand, plane tree leaves were from the plane tree, *Platanus orientalis* L. (known as *Platanaceae*). This type of leaves consists of carotenoids, unsaturated fatty acids, and terpenes [55]. Plane tree leaves create an environmental problem, according to the literature, because they can accumulate lead from car traffic exhaust [56].

**Figure 9:**
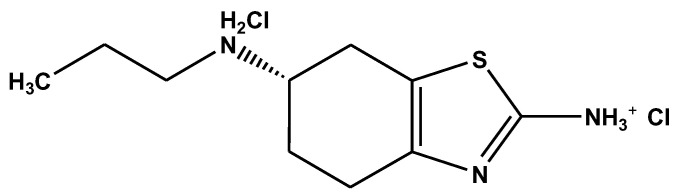
Schematic of the chemical structure of pramipexole dihydrochloride.

The pharmaceutical compound pramipexole dihydrochloride (C_10_H_21_Cl_2_N_3_OS; MW = 324.44 g/mol) was used as obtained from Amino Chemical Ltd. (Marsa, Malta). Figure 9 illustrates its chemical structure. Distilled water was used for the preparation of the solutions as well as for any process in this study.

Pramipexole dihydrochloride [(6S)-*N*6-propyl-4,5,6,7-tetrahydro-1,3-benzothiazole-2,6-diamine], a novel nonergoline dopamine agonist, was selected as the other model compound. Pramipexole was initially introduced for the treatment of the signs and symptoms of idiopathic Parkinson’s disease [57] and was recently approved in the US and Europe for the treatment of idiopathic restless legs syndrome in adults [58]. It is widely used all over the world for its unique pharmaceutical activity; on the basis of recent drug usage trends [59], it seems likely that the use of this recently available nonergot dopamine agonist will continue to increase in the immediate future as primary care physicians (PCPs) become more familiar with it. Some pharmaceutical industries and hospitals are discharging pramipexole in their effluents, resulting in the contamination of our natural water resources. It is important to note that until now, no data are given in the literature for the detection of pramipexole in water/wastewater. However, as described above, pramipexole has similar groups to other pharmaceutical compounds, so the study of its removal from aqueous systems can be a good tool for explanations. Additionally, the chemical agents used for the synthesis of ACs are ΝaOH and HCl; they were of analytical grade and purchased from Sigma-Aldrich, which is located in St. Louis, MO, USA.

### 3.2. Synthesis of Activated Carbon Samples

#### 3.2.1. PL(char)-AC and TEA(char)-AC

For the two-step synthesis of biomass-derived activated carbons (ACs), three district processes were followed: (i) carbonization, (ii) impregnation, and (iii) activation. Briefly, 10 g of tea leaves were inserted in a porcelain boat and carbonized at 650 °C (heating rate 10 °C/min) for 1.5 h under constant N_2_ (99.9% pure), with 30 cm^3^/min (STP) flow. The yielded biochar was impregnated with NaOH (3 M) aqueous solution under sonication for 2 h, followed by magnetic stirring for 24 h at 70 °C. Afterward, the synthesized solution was filtered in order to separate the impregnated biochar, and the obtained solid part was placed in an oven and dried at 110 °C for 24 h. The second activation process was carried out in a muffle oven, where the impregnated biomass was thermally treated again at 650 °C (heating rate 10 °C/min) for 2 h under the same N_2_ flow. After the process of pyrolysis, the synthesized activated carbons were ground with mesh screen 140 (105 μm) before their addition in the liquid phase (washing or adsorption process). After the cooling of the solid residue at ambient temperature, it was rinsed with HCl (37%) solution to remove the excess of NaOH and impurities and then with distilled water until neutral pH. Finally, the obtained material was dried at 110 °C for 24 h to obtain the final material, referred to as TEA(char)-AC. In the case of PL(char)-AC synthesis, the procedure was the same, except using plane tree leaves instead of tea leaves.

#### 3.2.2. TEA-AC

For one-step synthesis, the tea leaves (10 g) were placed in a vial containing a NaOH (3 M) aqueous solution under stirring for 24 h at 70 °C. Next, after filtration, the impregnated material was placed in an oven and dried at 110 °C for 24 h. The carbonization/activation process was carried out as in the second step of PL(char)-AC synthesis. The final obtained material was dried further at 110 °C for 24 h, and it is referred to as TEA-AC.

### 3.3. Adsorption Evaluation

#### 3.3.1. Effect of the Initial pH

The effect of the initial pH was studied by batch experiments. Briefly, 0.02 g of the AC sample (1 g/L) was inserted into 20 mL of PRM solution (C_0_ = 200 mg/L) in a conical flask. The adjustment of solution pH (at pH = 3, 5, 7, 9, and 11) was conducted with micro-additions of HCl (0.01 M) or NaOH (0.01 M) aqueous solutions. The flasks were allowed to agitate (160 rpm) at 25 °C for 24 h using a shaking bath. Finally, residual PRM concentration analysis was carried out. All experiments were conducted in triplicates.

#### 3.3.2. Isotherms

A weighted amount of AC samples (0.02 g) was inserted into a conical flask that contained 20 mL of PRM solution of different initial concentrations (C_0_ = 5–250 mg/L) at an initial pH of 3 (since the optimum value of the initial pH was found to be 3; from Section 3.3.1). The adjustment of initial pH was achieved with microadditions of HCl solution (0.01 M) or NaOH (0.01 M). Then, the flasks were shaken for 24 h at 25 °C with fixed agitation speed (160 rpm), using a shaking bath.

#### 3.3.3. Effect of Contact Time

A weighted amount of AC samples (0.02 g) was inserted into a conical flask that contained 20 mL of PRM solution of different initial concentrations (C_0_ = 200 mg/L) at an initial pH of 3 (since the optimum value of the initial pH was found to be 3; from Section 3.3.1). The adjustment of initial pH was achieved with microadditions of HCl solution (0.01 M) or NaOH (0.01 M). Then, the flasks were shaken for 24 h at 25 °C with fixed agitation speed (160 rpm), using a shaking bath. The residual concentrations of the ions were analyzed at predefined time intervals (5 min–24 h). Moreover, the calculations of residual concentrations of the ions (C_e_) in the liquid phase were achieved according to the section on “pH-effect”.

#### 3.3.4. Analysis and Modeling

Residual PRM concentration was evaluated using a UV spectrophotometer (U-2000, Hitachi, Tokyo, Japan). UV absorbance was adjusted to λ_max_ = 263 nm. The absorbance wavelength change was calculated from the obtained results, revealing an absorbance maximum change of ~2% and, hence, was determined as unimportant. The design of calibration curves from absorbance versus PRM concentration was achieved using the linear relationship of Beer–Lambert. In addition, using the equation of mass balance, as depicted below (Equation (1)), we calculated the PRM amount that was removed due to equilibrium phase Q_e_ (mg/g), where C_0_ and C_e_ (mg/L) are mentioned as the initial and equilibrium concentrations of PRM, respectively; V (L) is the aqueous solution volume; m (g) is the AC mass used.

(1) $$Q_{e}=\frac{\left(C_{0}-C_{e}\right)V}{m}$$

The experimental data from the equilibrium phase were fitted to the isotherm equations of Langmuir (Equation (2)) [60] and Freundlich (Equation (3)) [61], which are given by the below equations:

(2) $$Q_{e}=\frac{Q_{m}K_{L}C_{e}}{1+K_{L}C_{e}}$$

(3) $$Q_{e}=K_{F}C_{e}{}^{1/n}$$

where Q_m_ (mg/g) is the highest amount of adsorption; K_L_ (L/mg) is the Langmuir adsorption equilibrium constant; K_F_ (mg^1−1/n^ L^1/n^/g) is the Freundlich constant representing adsorption capacity; n (dimensionless) is the constant depicting adsorption intensity.

The experimental kinetic data fitting was achieved by using two widely known kinetic equations; (i) the pseudo first-order equation [62] (Equation (4)), and (ii) the pseudo second-order equation [63,64,65] (Equation (5)):

(4) $$C_{t}=C_{0}-(C_{0}-C_{e})\left(1-e^{-k_{1}t}\right)$$

(5) $$C_{t}=C_{0}-(C_{0}-C_{e})\left(1-\frac{1}{1+k_{2}t}\right)$$

where C_t_ (mg/L), k_1_ (min^−1^) and k_2_ (g mg^–1^ min^–1^**)** are the concentrations of PRM at specific time intervals and the kinetic constants derived from pseudo first-order and pseudo second-order equations, respectively.

*Nonlinear regression*. Sorption isotherm model parameters can be conveniently estimated via the nonlinear regression method using the original form of the isotherm equation. This method uses the same objective as described for the conventional linear regression method, which is to obtain model parameter estimates by minimizing the squared sum of the difference between experimental data and model outputs. It, however, differs from linear regression in that it is an iterative process [66]. It involves entering the experimental data first, in this case, equilibrium concentration C_e_ and amount of dyes adsorbed at equilibrium Q_e_ (for sorption equilibrium) and the time and amount of PRM adsorbed at time Q_t_ (for sorption kinetics), into the Excel spreadsheet and graphing the data. Initial estimates of the unknown parameters in the model equations are then made to calculate the theoretical Q_e_ or Q_t_ values from which the squared sum of the difference (SS) between the experimental data and the model (theoretical) output is obtained. Successive iterations are then performed, which involve changing the initial estimated parameter values by a small amount and recalculating the SS several times until the parameter values result in the lowest possible value of SS. Linear regression, on the contrary, usually requires only a single calculation to provide the lowest value of the SS. Nonlinear regression analysis is conveniently carried out using the Solver add-in function of the Microsoft Excel spreadsheet. Recently, Tran et al. [67] reviewed the inconsistencies of linearized forms of different isotherm models and their negative impact on the parameter values involved in the liquid-phase adsorption process. In general, nonlinear regression gives a more appropriate and accurate determination of model parameters than the linear regression method [68]. A detailed description of how to configure a spreadsheet for nonlinear regression using the Solver function is described elsewhere [31].

### 3.4. Characterization Techniques

The morphology of the synthesized ACs was evaluated via electron imaging using scanning electron microscopy (SEM, Jeol JSM-6390 LV, Tokyo, Japan). The value of accelerating voltage was adjusted to 15.00 kV, and the process of scanning was conducted in situ on a dry powder of AC. Surface functional groups were evaluated using an FTIR spectrometer (Perkin-Elmer FT-IR/NIR Spectrometer Frontier, Waltham, MA, USA) after 32 scans at a resolution of 2 cm^−1^, with baseline correction. In the apparatus used, the process is rapid and efficient without the need for KBr pellets, where the sample (powder) is placed onto the optically dense crystal of FTIR, and then a pressure tower applies force in order to press the sample. This apparatus offers spectra in the near, near-mid, mid, mid–far, and far infrared regions.

The specific surface areas were determined at 77 K via nitrogen adsorption/desorption tests using a Quantachrome analyzer (Nova 4200e, Boynton Beach, FL, USA) based on the Brumauer–Emmett–Teller (BET) model. Relative pressure (p/p^0^) was adjusted at approximately 0.005 to 0.985. Prior to the measurements of gas adsorption, the activated carbon was degassed at 200 °C in vacuum conditions of 0.1 torr for 24 h. The BET surface area was measured via the standard BET equation at a relative pressure of approximately 0.06 to 0.3. The total volume of pores was determined at approximately 0.985 (relative pressure) in order to make sure that all pores will be completely filled with N_2_ gas [50]. DFT pore size distribution and total pore volume for all cases of activated carbons were obtained from the N_2_ adsorption isotherms via the software provided by the manufacturer, which uses the BJH theory. The calculation of external surface area and micropore volume was achieved with the t-plot method. The micropore surface area was determined by difference [51].

## 4. Conclusions

The activated carbons derived from agricultural wastes (tea and plane tree leaves) have good adsorption capacity (Q_max_) for the removal of pramipexole dihydrochloride from aqueous solutions. It can also be concluded that carbonization/activation synthesis plays a key role since the ultrasound-assisted two-step protocol led to better-performing activated carbons. Additionally, the tea-leaf-derived material showed the highest RPM capacity of 112 mg/g at optimum pH = 3, a value higher than all the rest of the biomass-derived carbons reported in the literature. All the herein-studied activated carbons showed a bimodal microporous and mesoporous nature, with the tea leaves derived by the two-step protocol having the biggest amount of small mesopores and micropores. Consequently, according to the obtained results, the synthesized activated carbons derived from tea leaves and plane tree leaves can be used for the removal of pramipexole dihydrochloride from a liquid phase, and further surface chemistry modification can lead to even higher remediation efficiencies since the correlation of textural features (surface area) to adsorption capacity has been obtained.

## Figures and Tables

**Table 1:** Details of textural parameters of yielded ACs at activation temperature 650 °C.

Parameters	TEA(char)-AC	PL(char)-AC	TEA-AC
Surface area (m^2^/g)	1151	1105	594
Micropore surface area (m^2^/g)	774	508	168
External surface area (m^2^/g)	376.7	596.6	425.7
Total Pore volume (cm^3^/g)	0.694	0.884	1.782
Micropore volume (cm^3^/g)	0.369	0.236	0.078
Mesopore volume(cm^3^/g)	0.325	0.649	1.703
Ratio of micro- to mesopores volume	1.14	0.36	0.05

**Table 2:** Equilibrium parameters for the adsorption of PRM onto AC samples.

	Langmuir Equation			Freundlich Equation		
	Q_m_	K_L_	R^2^	K_F_	n	R^2^
Material	mg/g	L/mg		mg^1−1/n^ L^1/n^ g^−1^		
TEA(char)-AC	112	0.091	0.969	23.19	3.085	0.847
PL(char)-AC	101	0.063	0.962	18.16	2.763	0.846
TEA-AC	34	0.004	0.917	0.83	1.383	0.952

**Table 3:** Pseudo first-order and pseudo second-order equations for PRM uptake from aqueous solutions.

	Pseudo First-Order		Pseudo Second-Order	
	k_1_	R^2^	k_2_	R^2^
Material	(min^−1^)		(g mg^−1^ min^−1^)	
TEA(char)-AC	0.1255	0.994	0.1422	0.970
PL(char)-AC	0.0622	0.963	0.1294	0.980
TEA-AC	0.0713	0.994	0.2838	0.946

**Table 4:** Maximum adsorption capacities of different adsorbent materials for the removal of pramipexole from aqueous solutions.

Adsorbent	Q_max_ (mg/g)	Reference
Sulfonate grafted chitosan	337	[53]
*N*-(2-carboxybenzyl) grafted chitosan	307	[53]
Chitosan	181	[53]
Activated carbons (BAX)	117	[45]
Activated carbon from potato peels (hydrothermal)	66	[43]
Activated carbon from potato peels (pyrolized)	56	[43]
TEA(char)-AC	112	This study
PL(char)-AC	101	This study
TEA-AC	34	This study

## Data Availability

The data presented in this study are available upon request from the corresponding author.
